# Efficacy and Safety of Quetiapine for Pediatric Bipolar Depression: A Systematic Review of Randomized Clinical Trials

**DOI:** 10.7759/cureus.8407

**Published:** 2020-06-02

**Authors:** Sushma Srinivas, Tarun Parvataneni, Ramkrishna Makani, Rikinkumar S Patel

**Affiliations:** 1 Psychiatry, A.J. Institute of Medical Sciences and Research Centre, Mangalore, IND; 2 Psychiatry, Siddavanahalli Nijalingappa Medical College and HSK Hospital and Research Centre, Bagalkot, IND; 3 Psychiatry, AtlantiCare Health System, Egg Harbor Township, USA; 4 Psychiatry, Griffin Memorial Hospital, Norman, USA

**Keywords:** quetiapine, bipolar disorders, bipolar depression, child and adolescent psychiatry, clinical trial, efficacy

## Abstract

Quetiapine is a second-generation antipsychotic (SGA) approved by the Food and Drug Administration (FDA) for the treatment of schizophrenia, mania, and aggression in children and adolescents. It is also commonly used as an off-label medication to treat children and adolescents with bipolar depression, although the FDA has not approved quetiapine for this purpose. We conducted a systematic review of randomized clinical trials (RCTs) using the MEDLINE database and included two studies that met our inclusion criteria. Both RCTs were eight-week short-term studies that involved patients of 10-18 years of age with a Diagnostic and Statistical Manual of Mental Disorders-IV (DSM-IV) diagnosis of bipolar disorder, depressed type. The mean difference in the Children's Depression Rating Scale-Revised (CDRS-R) score and the response and remission rates in the quetiapine group were not statistically significant when compared to the placebo group. A high placebo response rate proved that quetiapine was no better than the placebo in treating pediatric bipolar depression. Quetiapine proved to be a relatively safe drug with the most common side effects being headache, somnolence, gastric upset, and weight gain. There was a significant increase in triglyceride levels, but no other metabolic effects were reported. This calls for future studies with larger sample sizes and improved methodology to explore the efficacy of quetiapine and other SGAs for the management of pediatric bipolar depression.

## Introduction and background

Bipolar disorder is characterized by mood dysregulation ranging from hypomania or mania to depression, along with unusual shifts in energy, activity levels, concentration, and the ability to carry out daily activities [1]. Approximately 1.8% of children and adolescents suffer from bipolar disorder [2]. Unlike adults, adolescents often present with unipolar or bipolar depression rather than manic episodes. This is often associated with suicidal ideation, aggression, or irritability. At least 50% of children presenting with unipolar depression go on to develop at least one episode of mania or hypomania before their adulthood, which qualifies them for the diagnosis of bipolar disorder [3]. It is hard to determine the exact incidence of bipolar depression, as most children develop manic symptoms that require treatment and hospitalization before the diagnosis of bipolar depression is made [4].

Since bipolar depression causes long-term effects and worsens the quality of life, it needs to be addressed and treated [5]. The available treatment modalities include both psychosocial (including cognitive behavioral therapy, dialectical behavioral therapy, and family therapy) and evidence-based pharmacological treatments such as selective serotonin reuptake inhibitors (SSRI), mood stabilizers (lithium, lamotrigine), and some second-generation antipsychotics (SGA) such as quetiapine, ziprasidone, and aripiprazole [5].

Quetiapine immediate-release (IR) and extended-release (XR) were approved by the Food and Drug Administration (FDA) in 2006 and 2008, respectively, for treating adults with bipolar depression [6,7]. It has been used as an off-label medication for bipolar depression in children, even though it is not FDA-approved in the pediatric population. While there have been a few randomized control trials (RCTs) conducted to study the efficacy of quetiapine in bipolar depression in children and adolescents, they have failed to establish the efficacy of the drug over placebo [8,9].

The goal of our systematic review was to perform a qualitative synthesis of past RCTs conducted on quetiapine in the pediatric population and to evaluate the overall efficacy and safety of quetiapine for managing bipolar depression.

## Review

Study search strategy and selection

**Figure 1:**
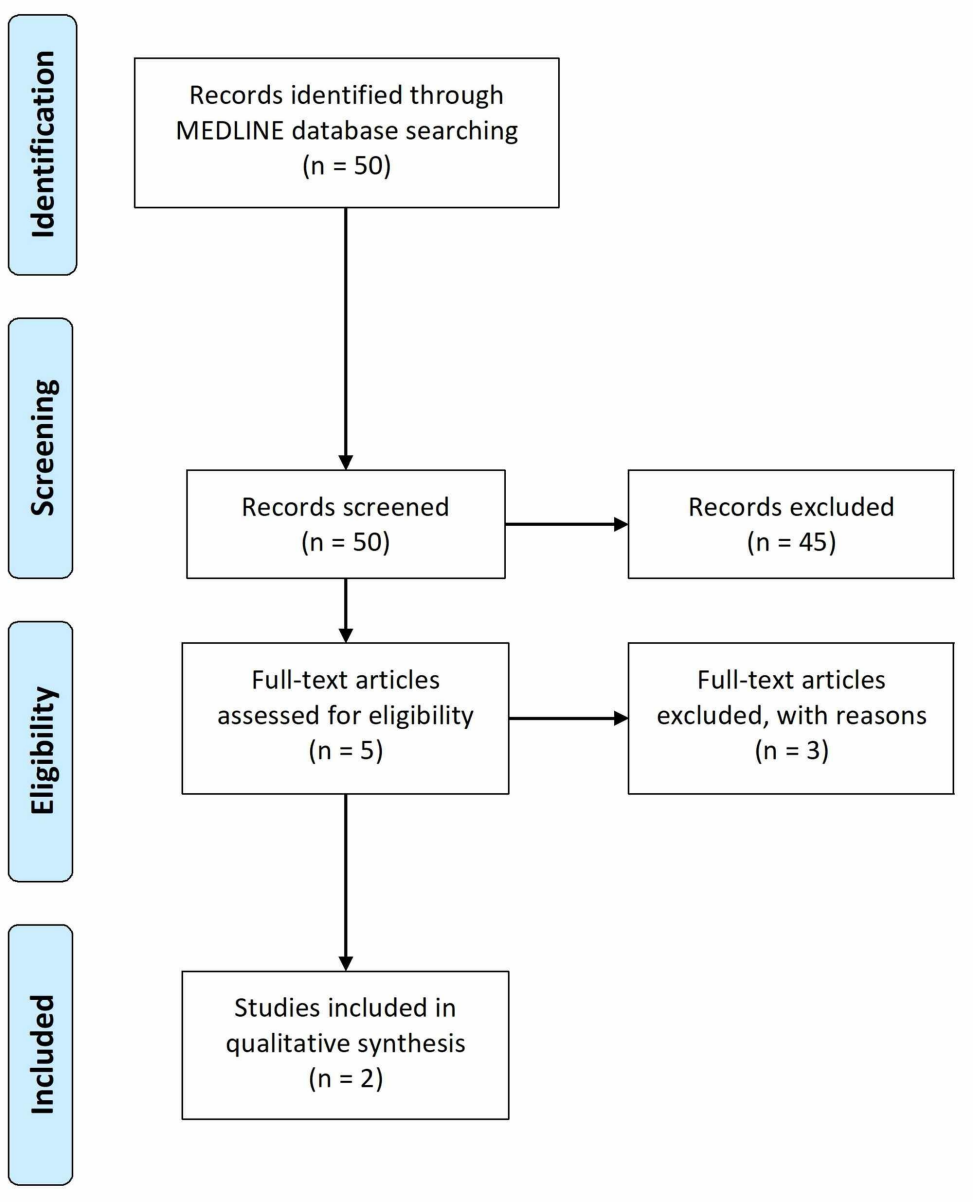
Results of the systematic review

The National Library of Medicine (NLM) MEDLINE database was used to identify clinical trials, clinical studies, multicenter studies, and observational studies published in English from March 31, 2010, to March 27, 2020, with the Medical Subject Headings (MeSH) terms "child" or "adolescent". The search strings "bipolar disorder", "bipolar", and "quetiapine" yielded 50 studies. All searches and screenings were done independently by two authors (R Patel and S Srinivas) using the Preferred Reporting Items for Systematic Reviews and Meta-Analyses (PRISMA) statement recommendations. The titles and abstracts were screened based on the purpose of our study objective and resulted in the inclusion of five studies. After reading the full texts, two studies met the criteria for our systematic review and were included in our final analysis, as shown in Figure 1.

Mechanism of action

Quetiapine is a low-affinity dopamine D2 and serotonin 5-HT2A antagonist. It is a partial agonist at the 5-HT1A receptor [7]. Quetiapine also acts as an alpha-1 and alpha-2 adrenergic receptor antagonist, which is responsible for unpleasant effects such as dizziness and orthostatic hypotension observed in some patients [10]. Its antagonist action on dopamine D2 receptor at the mesocortical and mesolimbic pathways makes it an effective antipsychotic to treat the symptoms of bipolar disorder and schizophrenia [10]. It also acts as an effective antidepressant through many mechanisms such as 5-HT1A agonism [7].

Pharmacokinetics

The active metabolite of quetiapine is norquetiapine, also known as N-desalkylquetiapine. It is generated by the cytochrome (CYP) 3A4 isoenzyme [11]. Norquetiapine inhibits the norepinephrine uptake, which is one of the mechanisms for the efficacy of quetiapine. The wide range of efficacy of quetiapine and norquetiapine is through dopamine D2, serotonergic, and noradrenergic neurotransmission [12].

Quetiapine is available in IR and XR formulations, with both having the same bioavailability. Higher plasma levels are sustained with the XR formulation, which has a longer duration of action, and thus is prescribed once daily and attains a peak plasma concentration at five hours. The IR formulation is short-acting and is prescribed twice daily, and attains its peak plasma concentration at two hours [13,14]. Quetiapine is metabolized and eliminated in the liver with a mean half-life of approximately six hours [10].

Pharmacodynamics

Quetiapine and its metabolite show a transiently high attachment to the dopamine D2 receptor and rapidly dissociates to very low levels by the end of the dosing interval [15]. It has minimal effect on the nigrostriatal and the tuberoinfundibular pathway and hence has a very low propensity to precipitate extrapyramidal symptoms (EPS) and hyperprolactinemia [16].

An increase in endogenous cytokines like interleukin-1B and interleukin-6 has been linked to depression [10]. By its antihistaminic activity, quetiapine indirectly downregulates the release of such endogenous cytokines, bringing about an antidepressant effect [10]. A decrease in dopamine neurotransmission in the prefrontal cortex is also linked to many symptoms of depression. Norquetiapine’s 5-HT2A and 5-HT2C antagonism inhibits the norepinephrine pathway, causing an increase in dopamine levels at the prefrontal cortex, thereby facilitating the antidepressant effect of quetiapine in mood disorders [13,17]. The partial 5-HT1A agonistic action of quetiapine (mainly norquetiapine) increases the serotonergic transmission at the raphe neurons of the brain stem and the limbic and cortical regions of the brain [13,18]. This also potentiates the antidepressant and anxiolytic effects of quetiapine. Norquetiapine’s 5-HT1A action also catalyzes neuron regeneration by increasing the release of a brain-derived neurotrophic factor in the hippocampus [13]. These are some of the mechanisms of action of quetiapine’s antidepressant effect.

Efficacy

The included double-blind, placebo-controlled (DBPC) RCTs were conducted in the pediatric population with bipolar depression for a period of eight weeks [8,9]. The primary efficacy measure was a >50% reduction in the Children's Depression Rating Scale-Revised (CDRS-R) score from the baseline to the endpoint (eight weeks). The secondary measures were the remission and the response rates [8,9].

DelBello et al. conducted a DBPC-RCT that included 44 adolescents aged 12-18 years with a Diagnostic and Statistical Manual of Mental Disorders-IV (DSM-IV) diagnosis of bipolar I disorder, depressed type, and a CDRS-R score of >40 [9]. The intervention group (n = 32) received 100 mg of quetiapine on day one, 300 mg on day three, with a flexible titration to 600 mg in the evening [9]. There was no statistically significant difference between the two groups, with the mean difference being -0.8 (p = 0.89). CDRS-R response and remission rates were 67% and 40% in the quetiapine group and 71% and 35% in the placebo group, respectively [9].

The main drawback of DelBello et al.'s DBPC-RCT was its small sample size, which limited the power of the study along with a high placebo response rate. This proved that quetiapine was not significantly efficacious than the placebo in reducing depressive symptoms [9]. So, Findling et al. used 150-300 mg of quetiapine, which is the approved dosage for treating bipolar depression in adults, and, to overcome the drawbacks found in DelBello et al. study, they used a larger sample size of 144 adolescents [8,19].

Findling et al. conducted an eight-week DBPC-RCT over 42 centers across seven countries. They included 144 adolescents aged 10-17 years with a DSM-IV diagnosis of bipolar I or II disorder, depressed type [8]. The intervention group (n = 70) received quetiapine XR ranging from 150 mg to 300 mg, starting with 50 mg on day one, 100 mg on day two, and 150 mg on day three [8]. If a clinical deterioration was observed in the second or third week on the clinical global impression scale for use in bipolar illness (CGI-BP-C of >5), the medication was increased in a stepwise manner to 300 mg [8]. The mean change in the CDRS-R score was -29.6 and -27.3 for quetiapine XR and placebo groups, respectively [8]. Response and remission rates were 63% and 45% for quetiapine XR and 55% and 34% for placebo groups, respectively, and there was no statistically significant difference between both groups (p = 0.25) [8].

The study by Findling et al. found a high placebo response rate of 55% despite an increase in sample size. Both the DBPC-RCTs found that the difference in CDRS-R score and the response and remission rates in the quetiapine group was not statistically significant when compared to the placebo group, thus concluding that quetiapine was no more effective than the placebo in improving the depressive symptoms in children and adolescents with bipolar depression [8,9].

The causes for the high placebo response rate noted in both DBPC-RCTs could be due to the small sample size, the disease severity at the baseline, the presence of other psychiatric comorbidities, the duration of the disease, and methodological factors like the number of study centers, the rating scales implemented, and the length of the lead-in period [8].

Adverse effects

Quetiapine was found to be a relatively safe drug with gastrointestinal upset, sedation, and dizziness being the most common complaints [8,9]. There were no reports of QTc prolongation of more than 420 milliseconds with statistically no significant differences between the quetiapine and placebo groups [9]. There was a substantial increase in triglyceride levels in the quetiapine group but non-significant changes in the other metabolic parameters and the EPS ratings [8,9].

A significant weight gain of 1.3 to 2.3 kg was seen in the quetiapine group compared to the placebo group [8,9]. Findling et al. reported that 12.5% of the quetiapine group and 6% of the placebo group had a more than a 7% increase in body weight [8]. Quetiapine acts on dopamine D2, 5-HT1A, and histamine receptors and releases brain neurochemicals that stimulate appetite and affect energy metabolism, leading to weight gain [8,9].

Regarding serious adverse effects, one patient in the quetiapine group had agitation, and one patient exhibited self-injurious behavior, although this was unrelated to the medication. Four patients in the placebo group had aggression and exacerbation of bipolar depressive symptoms [8]. In the study by DelBello et al., one patient in the quetiapine group attempted suicide by ingesting 16 quetiapine pills, leading to the removal of the patient from the RCT [9]. This patient had not been suicidal at baseline and had reported an improvement in the depressive symptoms, and her suicide attempt was precipitated by a psychosocial stressor [9]. No patients in the quetiapine group had suicidal ideation or attempts during the study period [8,9].

## Conclusions

Quetiapine is FDA-approved for treating manic episodes of bipolar disorder in the pediatric population (10-17 years of age) with a dose range of 400-800 mg/day. Although it is not yet approved for pediatric bipolar depression, it is commonly used as an off-label medication. Quetiapine is an overall well-tolerated medication with clinically significant metabolic adverse profile requiring meticulous screening for obesity, dyslipidemia, and hyperglycemia. There are some drawbacks in past DBPC-RCTs conducted to evaluate the efficacy of quetiapine for bipolar depression. This warrants more studies with larger sample size, head-to-head comparison with other psychotropic medications, and improved methodology to explore the efficacy of quetiapine and other SGAs for managing pediatric bipolar depression.
